# Supplementary material for: Antigen-based multiplex strategies to discriminate SARS-CoV-2 natural and vaccine induced immunity from seasonal human coronavirus humoral responses
Source: medRxiv. 2021 Feb 12:2021.02.10.21251518. Preprint. [Version 1] doi: 10.1101/2021.02.10.21251518 (PMC7885935; doi:10.1101/2021.02.10.21251518)
Supplement: Supplement 1 [file media-1.pdf]

SUPPLEMENTARY APPENDIX.

Antigen-based multiplex strategies to discriminate SARS-CoV-2 natural and vaccine induced immunity from seasonal human coronavirus humoral responses

Eric D. Laing[1]*, Spencer L. Sterling[1,2], Stephanie A. Richard[2,3], Nusrat J. Epsi[2,3], Si'Ana Coggins[1,2], Emily C. Samuels[1,2], Shreshta Phogat[1,2], Lianying Yan[1,2], Nicole Moreno[2,3], Christian L. Coles[2,3], Matthew Drew[4], Jennifer Mehalko[4], Caroline E. English[2,3], Scott Merritt[2,3,5], Katrin Mende[2,3,5], Vincent J. Munster[6], Emmie de Wit[6], Kevin K. Chung[7], Eugene V. Millar[2,3], David R. Tribble[3], Mark P. Simons[1,3], Simon D. Pollett[2,3], Brian K. Agan[2,3], Dominic Esposito[4], Charlotte Lanteri[2], G. Travis Clifton[5], Edward Mitre[1], Timothy H. Burgess[2], and Christopher C. Broder[1]*

Table S1. β-CoV MMIA performance

	SARS-CoV-2 PCR Status/Archival Sera			
SARS-CoV-2 spike IgM Antibody Test		Positive	Negative	Total
	Positive	110	0	110
	Negative	40	84	124
	Total	150	84	234
	Sensitivity	73.33%		
	Specificity		100.00%	

Table S2. IgG and IgM seropositivity within 28 days post-symptom onset (dpso)

dspo	IgG+	IgG+/IgM+
7 – 14	80.0% (12/15)	73.3% (11/15)
15 – 28	100% (31/31)	93.5% (29/31)

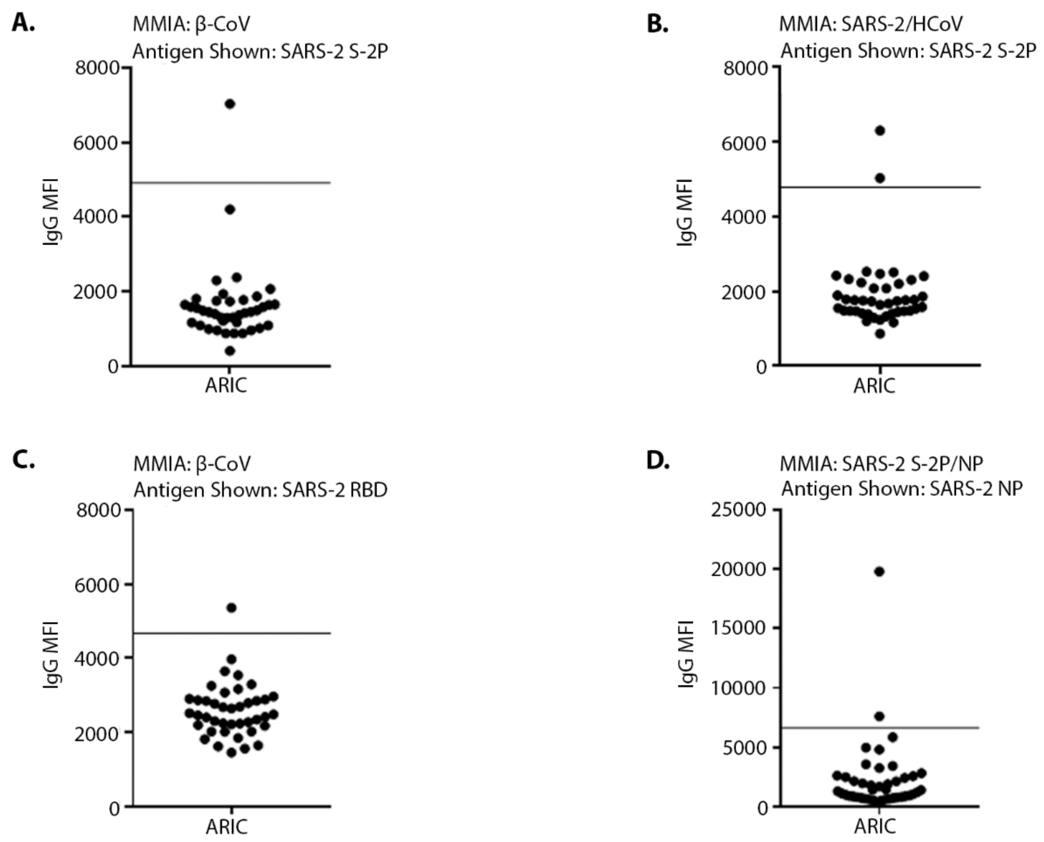

Figure S1. 99.7% probability distribution threshold cutoffs for SARS-CoV-2 antigen reactive IgG. Convalescent serum samples (n=43) from SARS-CoV-2 naïve ARIC subjects with HCoV PCR-confirmed infections were tested with MMIA indicated in panels. Antigens shown as follows: **(A)** SARS-CoV-2 spike (S-2P), **(B)** SARS-CoV-2 spike (S-2P), **(C)** SARS-CoV-2 RBD, and **(D)** SARS-CoV-2 NP; sold lines indicate the mean+3SD (99.7%) MFI.

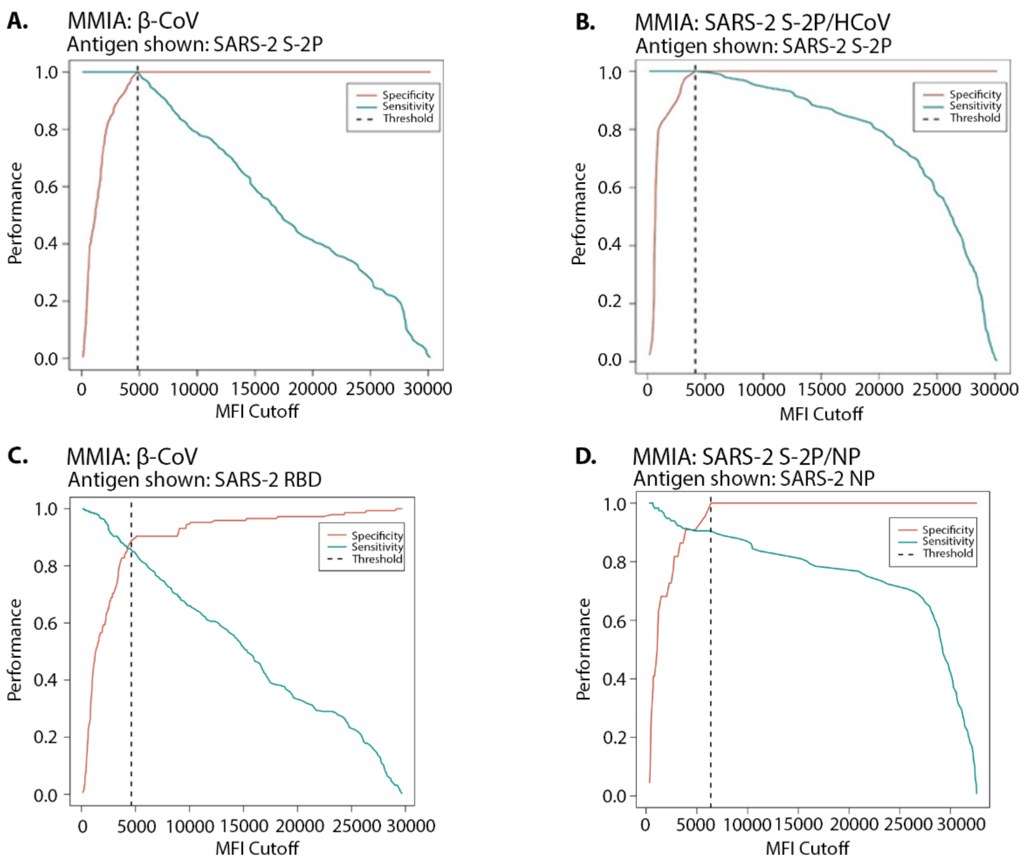

Figure S2. Receiver operating characteristic (ROC) curve analysis of SARS-CoV-2 PCR-positive and -negative serum samples from the EPICC study provided a second measurement of threshold cutoffs for SARS-CoV-2 antigen reactive IgG. EPICC Sera were tested with MMIA indicated in panels; antigens shown as follows: **(A)** SARS-CoV-2 spike (S-2P), **(B)** SARS-CoV-2 spike (S-2P), **(C)** SARS-CoV-2 RBD, and **(D)** SARS-CoV-2 N

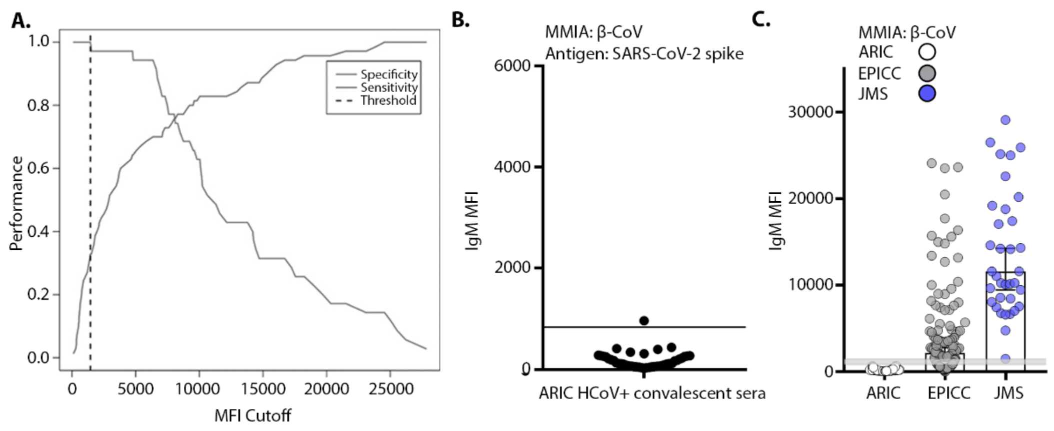

Figure S3. SARS-CoV-2 spike reactive IgM detection with the β-CoV MMIA.

(A) ROC curve analysis of SARS-CoV-2 spike protein IgM antibody reactivity in a β-CoV MMIA. PCR-confirmed SARS-CoV-2 positive and negative serum samples (n= 105) were tested and 100% specificity was achieved at threshold cutoff of 1446 MFI. **(B)** SARS-CoV-2 naïve ARIC HCoV PCR-positive convalescent serum samples (n=43) were tested to establish a 99.7% probability threshold (840 MFI). **(C)** ARIC (n=84), SARS-CoV-2 PCR-positive EPICC (n=116) and JMS (n=35) serum samples were tested in technical duplicates.
